# Mitigation of sepsis-induced liver injury by Clemastine via modulating GSDMD/NLRP-3/Caspase-1/NF-κB signalling pathways

**DOI:** 10.1186/s40001-025-02982-w

**Published:** 2025-08-06

**Authors:** Mahmoud Abdelnaser, Mina Ezzat Attya, Mahmoud A. El-Rehany, Moustafa Fathy

**Affiliations:** 1 Department of Biochemistry, Faculty of Pharmacy, Deraya University, Minia, 61111 Egypt; 2 Department of Pathology, Faculty of Medicine, Minia University, Minia, 61519 Egypt; 3 Department of Pathology, Faculty of Medicine, Minia National University, New Minia, 61111 Egypt; 4 Department of Biochemistry, Faculty of Pharmacy, Minia University, Minia, 61519 Egypt; 5 Department of Biochemistry, Faculty of Pharmacy, Minia National University, New Minia, 61111 Egypt

**Keywords:** Clemastine, Sepsis, Pyroptosis, NLRP-3, Caspase-1, GSDMD c-NT

## Abstract

**Aims:**

Nearly 48 million people have sepsis every year, and 11 million lose their lives as a direct consequence of the disease. In addition, sepsis is still the fifth leading death cause globally. The objective of this research was to find out whether pretreatment with Clemastine (CLM) would prevent septic liver damage.

**Main methods:**

Sepsis induction was established via CLP in male Wister rats. Histopathological analysis and hepatic function panel were assessed. The colorimetric method was used to assess hepatic contents of MDA, GSH, and SOD. ELISA was utilized to evaluate the hepatic TNF-α, IL-18, and IL-1β. qRT-PCR was utilized to evaluate *caspase-3, Bax, Bcl-2, and NF-kB* mRNA levels. Western blotting assessed NLRP-3, caspase-1, and GSDMD c-NT proteins.

**Key findings:**

CLP induced hepatic dysfunction, ALT and AST elevation, increased oxidative stress parameters, and escalated hepatic levels of TNF-α, IL-18, and IL-1β. It also augmented NLRP-3, caspase-1, and GSDMD c-NT protein levels, elevated Bax, NF-κB, and caspase-3 mRNA levels, and concurrently inhibited Bcl-2 mRNA levels. Conversely, CLM significantly mitigated molecular, biochemical, and histological changes induced by sepsis. CLM decreased proinflammatory signals, suppressed the production of NLRP-3, caspase-1, and GSDMD c-NT proteins, repressed *caspase-3, Bax,* and *NF-κB*, mRNA expression, and enhanced *Bcl-2* mRNA expression.

**Significance:**

Finally, by suppressing the NLRP-3/Caspase-1 mediated pyroptotic cell death in rats, CLM pretreatment provided protection against septic-liver damage.

## Introduction

Sepsis, a complex set of disorders, is typically induced by a systemic inflammatory reaction and results in progressive injury in various organs [1, 2]. Ultimately, in critical patients with serious illnesses, multiple organ failure is a common mortality cause and constitutes an urgent clinical situation [1, 3]. When compared to individuals without sepsis, sepsis survivors frequently experience more severe abnormalities and more significant readmission rates [4, 5]. Estimating the global effect of sepsis is difficult; nonetheless, recent scientific research estimated that in 2017, there were 48 million instances of sepsis and 11 million fatalities attributed to it yearly, accounting for about twenty percent of deaths around the world [6]. Sepsis may damage various organs, including the kidneys, brain, liver, lungs, and heart [5, 7, 8]. Surprisingly, the liver contributes to sepsis etiology and is an essential part of the host response that aids in the clearance of pathogens and other toxic substances during septic shock [9, 10]. Patients suffering from chronic liver disorders, including drug-caused liver damage and cirrhosis, have a higher sepsis risk, multiple organ damage, and sepsis-related death than individuals without intrinsic hepatic disorders. Liver failure and dysfunction produced by sepsis are closely related to a high death rate [11, 12]. The frequency of liver damage associated with sepsis varies from 34 to 46% [13, 14]. In early sepsis, for instance, liver failure often happens 90 min after cecal ligation and puncture (CLP) [15]. Scientists often evaluate the possible protective effects of various drugs using the CLP model of polymicrobial-induced sepsis, as it closely mimics clinical sepsis [16–18].

Pyroptosis, present in conditions of pathogenic infection and crucial in sepsis, is a necrotic cell death triggered by a cytosolic inflammasome, which causes inflammation [19, 20]. As members of the cytoplasmic multi-protein complex class, inflammasomes can recognize certain pathogen-associated molecular patterns (PAMPs) and danger-associated molecular patterns (DAMPs) that are activated during sepsis [21, 22]. The most extensively investigated canonical inflammasome is nucleotide-binding oligomerization domain (NOD)-like receptor protein 3 (NLRP-3), whose activation leads to caspase-1 activation [23, 24]. The gasdermin D (GSDMD) protein, the executioner of pyroptosis, is proteolyzed by active caspase-1 into active GSDMD, forming cell membrane pores [25, 26]. Caspase-1 activates and cleaves IL-1β and IL-18 precursors, resulting in the formation of mature IL-1β and IL-18 that are liberated through pores and cause inflammation [25]. Furthermore, numerous studies reported that pyroptosis is linked to sepsis incidence and development [27–30].

Currently, there are no therapies specifically approved for the treatment of sepsis-induced liver injury; management is largely supportive and focused on treating the underlying infection and maintaining organ function. Several pharmacological agents have been investigated for their potential to mitigate liver injury in sepsis, such as corticosteroids, which may reduce systemic inflammation and improve hemodynamics [31]. However, their benefit in preventing or treating liver injury is not well established, and prolonged use can increase the risk of secondary infections and metabolic complications. Moreover, an antioxidant and glutathione precursor, N-acetyl cysteine, has shown hepatoprotective effects in some experimental and clinical settings of acute liver injury, including sepsis [32]. Its advantages include a favorable safety profile and the ability to reduce oxidative stress; however, clinical evidence for efficacy in sepsis-induced liver dysfunction remains limited and inconclusive. So, researchers try to find potential safe medications with minimal adverse effects that might protect against sepsis-induced organ damage.

The first-generation antihistaminic drug Clemastine (CLM), which has a cholinergic blocking activity, is frequently indicated to treat different types of allergies. The approval of the FDA indicates that CLM was the most efficacious antimuscarinic medication that markedly improved oligodendrocyte formation [33].

This study is inspired by the evidence that CLM has been documented to protect against numerous organ injuries in different experimental models. CLM mitigated cardiac dysfunction caused by sepsis via regulating the Bax and Bcl-2 signals [34]. Additionally, CLM alleviated hypo-myelination brought on by brain damage resulting from hypoxic ischemia by reducing the IL-1 β and P38 levels [35].

Moreover, CLM alleviated autoimmune encephalomyelitis via regulating Nrf2/HO-1 and NLRP3 expression [36].

Sepsis after surgery is a prevalent and serious complication that may result in negative post-operative outcomes, prolonged periods of hospitalization, and mortality rates that may increase by up to 25 times [37]. Despite advancements in critical care during the last two decades, fatalities from postoperative peritonitis remain around fifty percent [38]. Additionally, we focused on mimicking the clinical situations in which some patients following surgery may be at risk for sepsis and sepsis-related hepatic dysfunction. Consequently, CLM may serve as an effective therapy for sepsis-induced hepatic dysfunction in addition to its documented cardioprotective positive outcomes in sepsis. Furthermore, administering CLM before surgery might provide further advantages by diminishing secretions and mitigating surgical operations-induced vagal bradycardia, explained by its antimuscarinic properties.

In light of the previously published results, the current study evaluated if CLM might prevent liver damage brought on by sepsis, for the first time, by preventing NLRP-3/Caspase-1 pathway-mediated pyroptotic cell death in rats.

## Materials and methods

### Drugs

Clemastine was acquired from GlaxoSmithKline, located in Brentford, United Kingdom. Vitamin C was provided by Eipico (Cairo, Egypt). Before administration, all medications were reconstituted in 0.9% NaCl.

### Ethical approval and guidelines

In compliance with the Faculty of Pharmacy Ethics Committee standards, Minia University, Egypt. The animal procedures were conducted with the authorization request designated by Approval ID: E S 07/2021. Furthermore, all animal experiments adhered to the National Institutes of Health guide for the care and use of Laboratory animals (NIH Publications No. 8023, revised 1978) and complied with the ARRIVE guidelines.

### Animal care

Adult male Wistar rats (n = 108) weighing between 200 and 220 g at 7–9 weeks of age were used in the investigation. They were acquired from Assiut University (Assiut, Egypt). The rats were housed in polypropylene enclosures under standardized laboratory circumstances for seven days before the beginning of the experiment. which involved a temperature of 25 ± 2 °C, a relative humidity of 50 ± 20%, and a light/dark cycle of 12 h. The rats were given a standard pellet diet and allowed unlimited water access.

### CLP induction

Sepsis was induced utilizing the reported CLP method [39]. In summary, the abdominal wall was subjected to shaving and subsequently disinfected with a 10% povidone-iodine solution. Following that, the rats were administered an intraperitoneal (i.p.) injection of a xylazine (10 mg/kg) and ketamine (100 mg/kg) mixture to enable the induction of anesthesia [40]. Subsequently, an incision was executed in the lower left quadrant of the abdomen. Following the exteriorization and ligation of the cecum utilizing 0.3-mm silk surgical suture thread, two punctures were created in the cecum with an 18-gauge hypodermic needle to establish a through-and-through perforation in the ligated region. The degree of sepsis was kept constant by ligating the cecum up to three quarters of its length. Without CLP, the same procedures and resuscitation approaches were used on rats having a sham operation.

### Study design

A total of 48 male Wistar rats were randomly divided into 6 groups, with 8 rats of each:

The first group (sham rats): Rats received a single i.p. injection of 0.5 ml of isotonic saline prior to the CLP surgical procedures, without undergoing CLP.

The second group (Clemastine 50): Rats received a single i.p. injection of CLM at a dosage of 50 mg/kg.

The third group (CLP): Rats received a single i.p. injection of 0.5 ml isotonic saline once prior to CLP procedure.

The fourth group (CLP + Clemastine 30): Rats received a single i.p. injection of CLM at a dosage of 30 mg/kg as a single dose prior to CLP procedure [34].

The fifth group (CLP + Clemastine 50): Rats received a single i.p. injection of CLM at a dosage of 50 mg/kg as a single dose prior to CLP procedure [34].

The sixth group (CLP + vitamin C): Rats received a single i.p injection of vitamin C (200 mg/kg) after the CLP procedure [18, 41].

The doses of CLM were selected based on a recent study that illustrated its antioxidant and anti-inflammatory properties in experimental models of sepsis-induced myocardial injury [34].

For each group, animal euthanization was conducted 24 h subsequent to CLP procedure.

The same previously mentioned groups of 60 rats (n = 10) were assigned at random to carry out the survival investigation. For ten days, the survival rates of every group were recorded.

Animals were randomly assigned to experimental groups using a computer-generated randomization schedule. This process was performed by a researcher not involved in the subsequent experimental procedures.

All outcome assessments, including histopathology, biochemical assays, ELISA, qRT-PCR, and Western blotting, were performed by investigators blinded to group allocation. Blinding was maintained until the completion of data analysis.

### Tissue isolation

On the second day, rats received urethane i.p. to produce anesthesia at a concentration of 25% at a dosage of 1.6 g/kg [42]. The abdominal aortas were used to get blood samples, then the rats were euthanized by a cervical dislocation method. Following 15 min of 4000 × g centrifugation, the serum was extracted and stored at −20 °C for additional biochemical tests. The liver tissues were immediately separated and rinsed with cold (10X) PBS, pH 7.4. Hepatic tissue specimens were divided into four segments. The initial portion was preserved at −20 °C following flash-freezing in liquid nitrogen, for additional biochemical analysis. Western blotting and subsequent quantitative real-time PCR (qRT-PCR) investigations were conducted on the second and third sections, which were preserved at −80 °C. The fourth fragment was preserved in 10% formaldehyde for histological analysis.

### Sepsis biomarker detection

The blood level of procalcitonin (PCT) was evaluated using a rat PCT ELISA kit (E-EL-R2400, Elabscience, Houston, Texas, USA) in accordance with the manufacturer's instructions based on the previously reported method [43].

### Hepatic function panel detection

The hepatic function panel was evaluated by serum ALT and AST evaluation kits (#1001171, #41270, respectively, SPINREACT, Spain) as directed by the manufacturer, based on the previously reported method [44].

### Oxidative stress parameters detection

Phosphate-buffered saline (10X) pH 7.4 (PBS) (AM9624, UK) was utilized for hepatic tissue homogenization. One to ten was the ratio of tissue weight to homogenization buffer. At 4 °C, the homogenates underwent a 10-min centrifugation run at 4000 × g. The obtained supernatant was used to determine the hepatic levels of malondialdehyde (MDA), reduced glutathione (GSH) and superoxide dismutase (SOD) (MD2529, GR2511, SD2521, respectively, Biodiagnositic, Cairo, Egypt) as directed by the manufacturer.

### Inflammatory cytokines detection

Interleukin-18 (IL-18), interleukin- β (IL- β), and tumor necrosis factor-alpha (TNF-α) hepatic tissue levels were assessed utilizing ELISA kits (E-EL-R0567, E-EL-R0012, E-EL-R0019, respectively, Elabscience, Texas, United States) as directed by the manufacturer, based on the previously reported method [43].

### Alpha klotho detection

The serum level of α-Klotho (KLA) was detected by a rat KLA ELISA kit (E1206Ra, BT LAB, Zhejiang, China) as directed by the manufacturer, based on the previously reported method [45].

### Quantitative real-time polymerase chain reaction

The expression of the genes Bcl-2-associated X (*Bax*), B-cell lymphoma-2 (*Bcl-2*), *NF-kB*, and *caspase-3* in the liver was investigated using real-time polymerase chain reaction (PCR).

Hepatic samples, each weighing 100 mg, were subjected to ultrasonic homogenization using 1 mL of TRIzol reagent, utilizing a Branson Digital Sonifer ultrasonic cell homogenizer (SFX 550, Danbury, USA). We evaluated the total RNA amount and used the A260/A280 ratio to test purity. RNA samples that met or above purity ratings of 1.8 were subjected to qRT-PCR. Total RNA was converted into complementary DNA (cDNA) in equal quantities utilizing the Revert Aid First Strand cDNA Synthesis Kit (K1622, Thermo Fisher Scientific, USA). qRT-PCR was conducted utilizing single-stranded cDNA. Table 1 depicts the sequences of the primers. Using Thermo Scientific Maxima SYBR Green qPCR Master Mix (2X) (K0251, Thermofisher Scientific, USA) the PCR procedure was performed by a Step One qRT-PCR Detection System (#4369074, Applied Biosystems, Singapore). The expression levels of each target gene were normalized to the expression of GAPDH, and then the 2 ^(–∆∆Ct)^ method was used to determine the levels of gene expression [46].

**Table 1:** The sequences of primers

Gene		Sequence (5'to 3')
*Bax*	F	CACGTCTGCGGGGAGTC
	R	TGTTGTCCAGTTCATCGCCA
*Bcl-2*	F	GGGCTACGAGTGGGATACTG
	R	GACCCCACCGAACTCAAAGA
*NF-kB*	F	CAGCAGATGGCCCATACCTT
	R	CTGTCATCCGTGCTTCCAGT
*Caspase-3*	F	GGAGCTTGGAACGCGAAGAA
	R	CCATTGCGAGCTGACATTCC
*GAPDH*	F	CTCTCTGCTCCTCCCTGTTC
	R	CGACATACTCAGCACCAGCA

### Western blotting analysis

The expression levels of NLRP-3, caspase-1, GSDMD c-NT, Phospho-NF-κB p65 and cleaved caspase-3 proteins were evaluated utilizing western blotting [47, 48].

RIPA Lysis buffer (89900, USA) was used to homogenize hepatic tissues. The protein concentration has been measured using the Bradford technique [49]. The homogenates, which contained 30 μg of total protein, were run through 12% sodium dodecyl sulfate–polyacrylamide gel electrophoresis (SDS-PAGE) for two hours at 100 V after being treated with loading buffer containing 2-mercaptoethanol for five minutes. After electrophoresis, the proteins transferred onto PVDF membranes were subjected to a blocking procedure for one hour utilizing a Tris-buffered saline (TBS-T) blocking solution, which involved 0.05 percent Tween-20 and 5% (w/v) non-fat milk. Electrophoresis and electroblotting were conducted utilizing a BioRad Transblot apparatus (Bio-Rad, CA, United States). The incubation with primary antibodies was performed overnight at 4 °C and included the following: anti-NLRP3 (ab263899, Abcam, UK), anti-Caspase-1 (ab138483, Abcam, UK), anti-cleaved N-terminal GSDMD (ab215203, Abcam, UK), anti NF-κB p65 Rabbit (#8242, cell signaling, USA), rabbit anti-Phospho-NF-κB p65 (##3033, cell signaling, USA), rabbit anti cleaved Caspase-3 (#9661, cell signaling, USA), and β-actin (#sc-47778, Santa Cruz, CA, USA). In a blocking buffer solution, a secondary antibody, specifically horseradish peroxidase (HRP)-conjugated polyclonal immunoglobulin at a dilution of 1:5000 (#7074, Cell Signaling, Massachusetts, USA), was utilized. To identify immunoreactive proteins, a luminous image analyzer (LAS-4000, Tokyo, Japan) in conjunction with a chemiluminescence kit (GE Healthcare, Little Chalfont, UK) was utilized as directed by the manufacturer. The Image J program (ImageJ, 1.8.0_172) was used to conduct densitometric analysis. For each blot, the background intensity was measured in an area adjacent to each band and subtracted from the band’s raw intensity value to correct for non-specific signal. The intensity of each target protein band was normalized to the corresponding β-actin band from the same lane.

The normalized value was calculated as:

Relative Expression = (Target Band Intensity – Background)/(β-Actin Band Intensity – Background).

All data were then expressed as a fold change relative to the sham control group.

### Histopathological analysis of the hepatic tissues

Before assessing the histological alterations in the liver, the liver was first fixed for one day in a 10% formalin solution. The liver was then sectioned using a microtome and stained with hematoxylin and eosin (H&E) [50]. Subsequently, the slides were analyzed, and digitally magnified photos were captured using a microscope (Olympus, Tokyo, Japan), which included a high-quality digital camera. Evaluation and blind observation were performed in a random order. A semi-quantitative analysis was carried out using a scoring methodology based on criteria such as nuclear necrosis, hepatocyte ballooning, inflammatory infiltration, and vascular congestion. For every slide of the liver tissue section, every parameter was rated, and the findings were presented as the aggregate of the individual score grades, ranging from normal (0), mild (1), moderate (2), and severe (3) [51]. Moreover, the histological semi-quantification of focal inflammatory cell infiltration in liver tissue was performed blindly for 5 different fields of a section, and the mean of the number of focal inflammatory cell infiltrations was calculated as follows:

0 (Normal): None, 1 (Mild): < 2 foci/100 × field, 2 (Moderate): 2–4 foci/100 × field, and 3 (Severe): > 4 foci/100 × field [52]. Additionally, scores were used to express nuclear necrosis, hepatocyte ballooning, and vascular congestion as follows: 0; normal, 1; mild (< 10% of hepatocytes in the centrilobular area), 2; moderate (10–50% of hepatocytes in the centrilobular area), 3; severe (> 50% of hepatocytes in the centrilobular area) [53].

### Statistical analysis

The mean ± standard deviation (SD) was used to present all the data. The Shapiro–Wilk test was performed to test the normality of data, and the data were normally distributed because the p-value of all parameters was more than 0.05. As the data were normally distributed, a one-way analysis of variance (ANOVA) was used to analyze the results, followed by the Tukey–Kramer test to compare the significance difference between groups. GraphPad Prism® was utilized to perform statistical analysis. A p-value of less than 0.05 is regarded as indicative of a statistically significant result. Moreover, the statistical difference of the survival study was conducted using the log-rank test.

## Results

### Clemastine improved the survival of septic rats

**Fig. 1:**
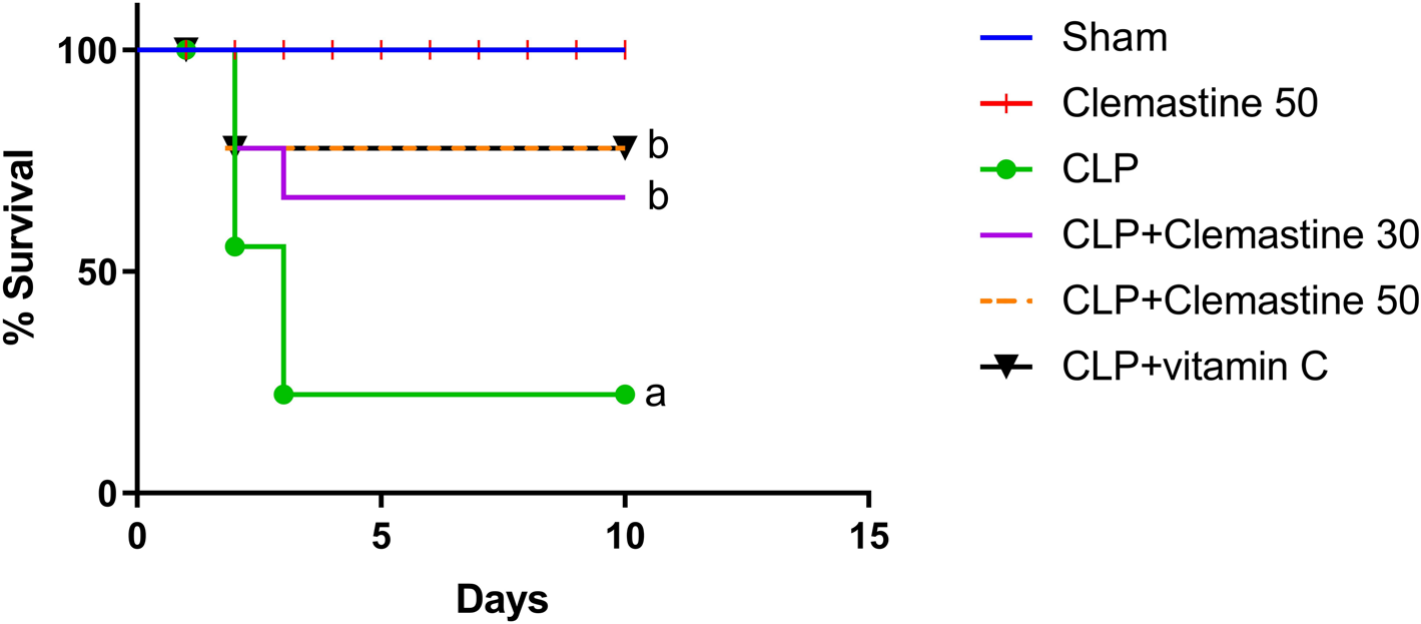
Clemastine effect on mortality induced by CLP (n = 10). The results indicated a significant difference, with a p-value of < 0.05 in contrast to sham rats (denoted as a) and a p-value of < 0.05 in contrast to CLP rats (denoted as b). The analysis was conducted using the log-rank test

Figure 1 shows that over the 10-day observation period, no fatalities occurred in the sham-operated group, however, 45% of the CLP rats died during the second day after surgery. In addition, 30% of CLP group died by day three. Conversely, high dose CLM treatment exhibited 20% mortality on the second day After CLP. In contrast to the untreated rats, which exhibited an overall survival rate of 25%, both CLM and vitamin C demonstrated a substantially higher overall survival rate of 80% (p < 0.05).

### Clemastine attenuated sepsis biomarker: PCT

**Fig. 2:**
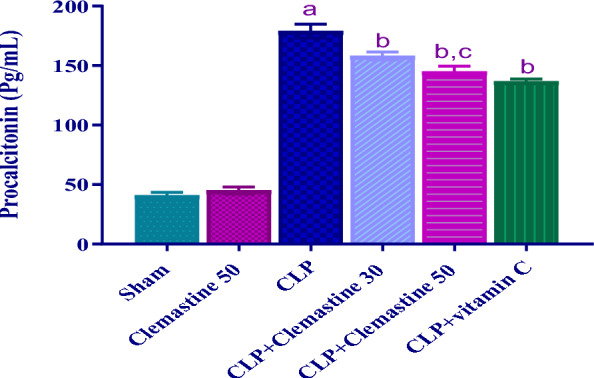
Clemastine effect on procalcitonin level in septic liver damage. Each value is represented as the mean ± standard deviation. (n = 6). Level of significance: a p < 0.05 in contrast to sham rats, b p < 0.05 in contrast to CLP rats, and c p < 0.05 in contrast to CLP + Clemastine 30

To determine CLM's potential protective role against sepsis, serum PCT level was evaluated. The PCT level after CLP was significantly upsurged (p < 0.05) relative to that observed in the sham rats, as depicted in Fig. 2. CLM and vitamin C administration revealed a marked decrease (p < 0.05) in PCT levels compared to the CLP rats. Interestingly, in contrast to CLP + Clemastine 30, the PCT level substantially declined (p < 0.05) in the CLP + Clemastine 50 group, as demonstrated in Fig. 2.

### Clemastine reduced hepatic function enzymes: ALT & AST

**Fig. 3:**
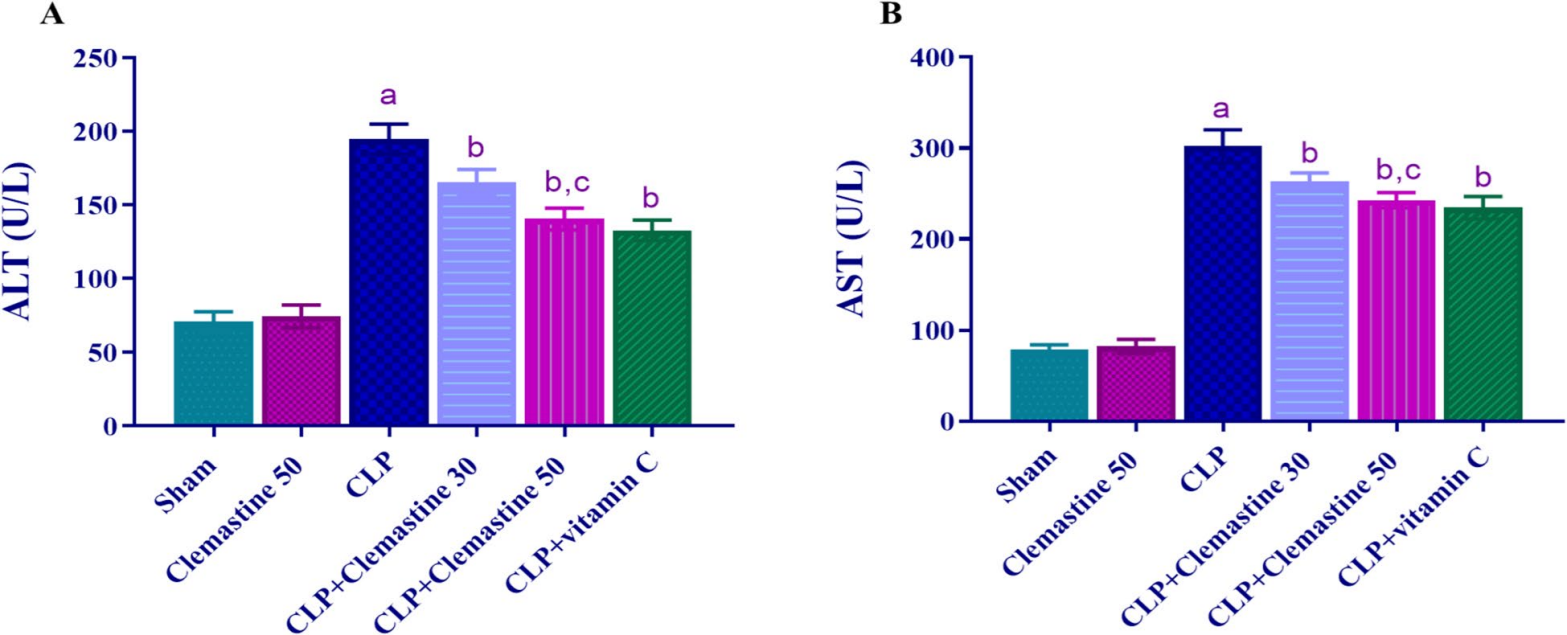
Clemastine effect on hepatic function panel in septic liver damage. **A** ALT activity, **B** AST activity. Each value is represented as the mean ± standard deviation. (n = 6). Level of significance: a; p < 0.05 in contrast to sham rats, b; p < 0.05 in contrast to CLP rats, and c; p < 0.05 in contrast to CLP + Clemastine 30. *ALT* alanine transaminase, *AST* aspartate transaminase

To evaluate CLM's potential protective role against hepatic damage in sepsis, serum ALT and AST activities were assessed. The CLP group revealed a dramatic increase (p < 0.05) in both ALT and AST activities relative to the sham rats, as observed in Fig. 3A, B. On the other hand, ALT and AST activities in the rats treated with CLM and vitamin C (used as a positive control) were dramatically lower (p < 0.05) than in CLP rats. Notably, CLM high dose demonstrated a marked decline (p < 0.05) in ALT and AST serum activities relative to the low dose, as depicted in Fig. 3A, B.

### Clemastine mitigated hepatic MDA

**Fig. 4:**
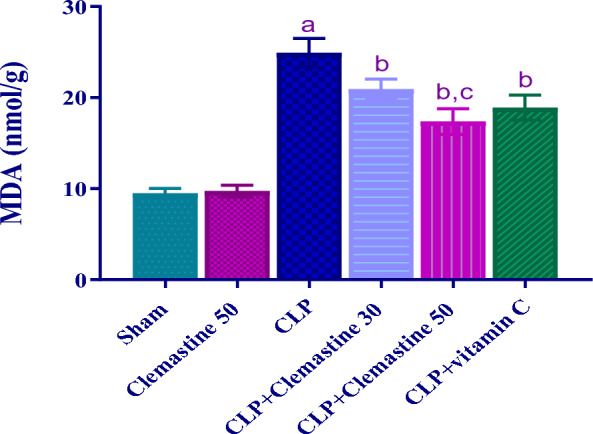
Clemastine effect on oxidative stress marker (MDA) in septic liver damage. Each value is represented as the mean ± standard deviation. (n = 6). Level of significance: a; p < 0.05 in contrast to sham rats, b; p < 0.05 in contrast to CLP rats, and c; p < 0.05 in contrast to CLP + Clemastine 30. MDA: malondialdehyde

Figure 4 demonstrates the liver tissue levels of MDA in different groups. In comparison to the sham rats, the MDA contents in the CLP rats were considerably higher (p < 0.05). In contrast, MDA levels in rats treated with CLM were dramatically lower (p < 0.05) than in the CLP rats. As observed in Fig. 4, the MDA levels in the CLP + Clemastine 50 group were considerably lower (p < 0.05) than those in the CLP + Clemastine 30 group.

### Clemastine improved antioxidant markers: SOD & GSH

**Fig. 5:**
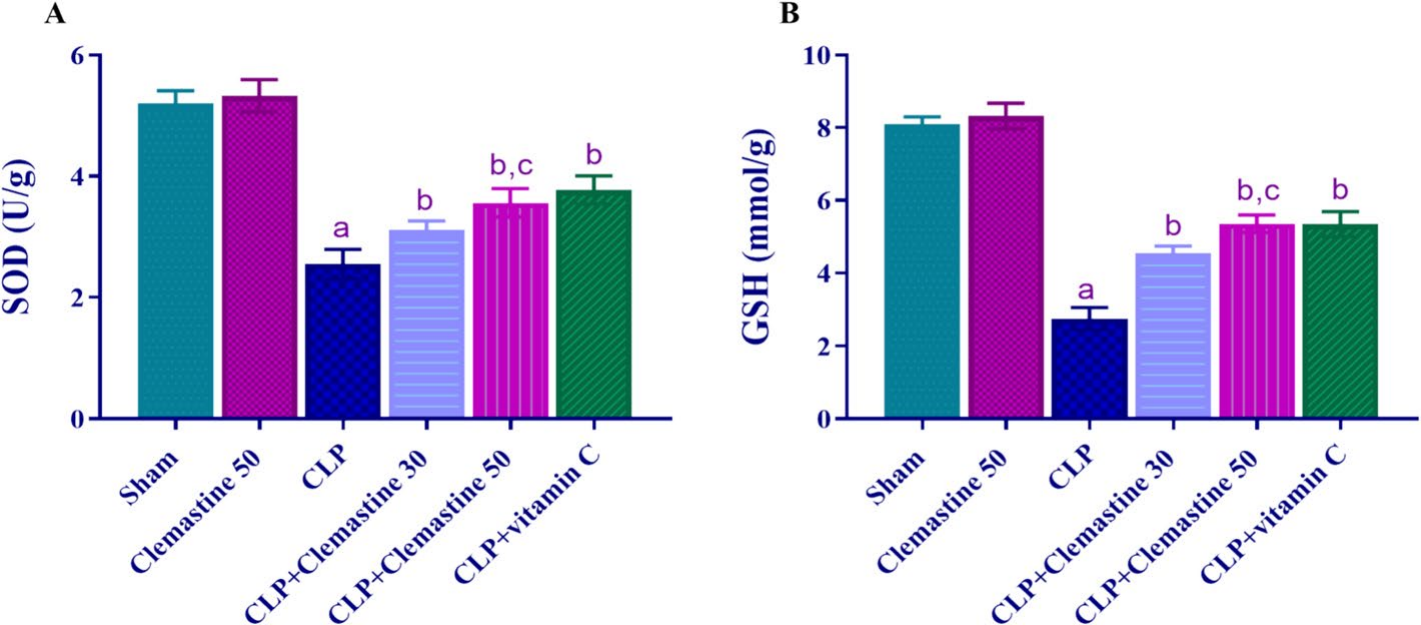
Clemastine effect on hepatic antioxidant defense markers in septic liver damage. **A** SOD activity & **B** GSH levels. Each value is represented as the mean ± standard deviation. (n = 6). Level of significance: a; p < 0.05 in contrast to sham rats, b; p < 0.05 in contrast to CLP rats, and c; p < 0.05 in contrast to CLP + Clemastine 30. SOD: superoxide dismutase, GSH: reduced glutathione

The hepatic concentrations of GSH and SOD were significantly reduced (p < 0.05) in the CLP rats, as illustrated in Fig. 5A, B, in comparison to the sham rats. Treatment with the two doses of CLM significantly reduced (p < 0.05) these changes compared to the CLP group. Notably, in contrast to the low dosage of CLM, the high dose demonstrated a significant increase (p < 0.05) in SOD and GSH levels.

### Clemastine inhibited hepatic cytokines: IL-18 & IL-1β & TNF-α

**Fig. 6:**
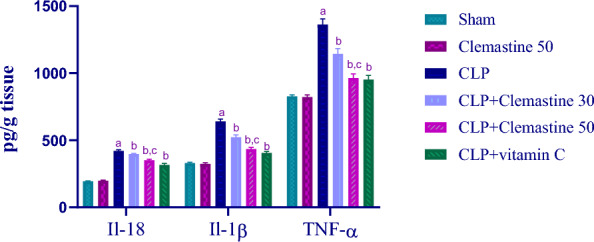
Clemastine effect on hepatic proinflammatory cytokines in septic liver damage. IL-18 & IL-1β & TNF-α levels. Each value is represented as the mean ± standard deviation. (n = 6). Level of significance: a; p < 0.05 in contrast to sham rats, b; p < 0.05 in contrast to CLP rats, and c; p < 0.05 in contrast to CLP + Clemastine 30. IL-18: interleukin 18, IL-1β: interleukin 1 beta, TNF-α: tumor necrosis factor-alpha

Figure 6 indicates that CLP significantly elevated (p < 0.05) hepatic tissue concentrations of pro-inflammatory cytokines (IL-18, IL-1β, and TNF-α) compared to the sham group. However, rats administered CLM exhibited a significant reduction (p < 0.05) in their liver levels in a dose-dependent pattern.

### Clemastine enhanced serum levels of KLA

**Fig. 7:**
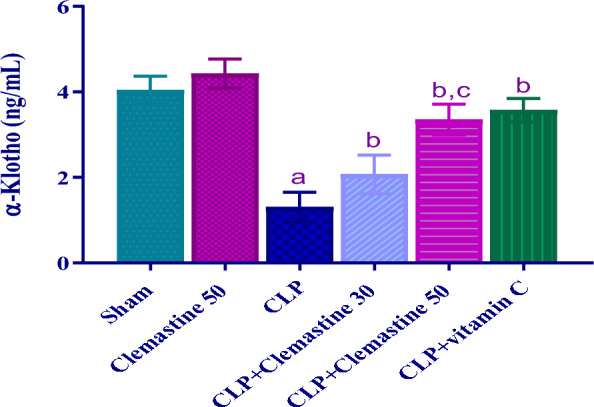
Clemastine effect on α-Klotho serum level in septic liver damage. Each value is represented as the mean ± standard deviation. (n = 6). Level of significance: a; p < 0.05 in contrast to sham rats, b; p < 0.05 in contrast to CLP rats, and c; p < 0.05 in contrast to CLP + Clemastine 30

Serum KLA level in the CLP rats was substantially (p < 0.05) lower than in the sham rats, as Fig. 7 demonstrates. Conversely, The KLA levels in rats treated with CLM showed a marked upregulation (p < 0.05) in contrast to the CLP rats. Surprisingly, the CLP + Clemastine 50 group revealed a considerable incline (p < 0.05) in the KLA level versus CLP + Clemastine 30.

### Clemastine ameliorated the *Bax* and* Bcl-2* gene expression imbalance

**Fig. 8:**
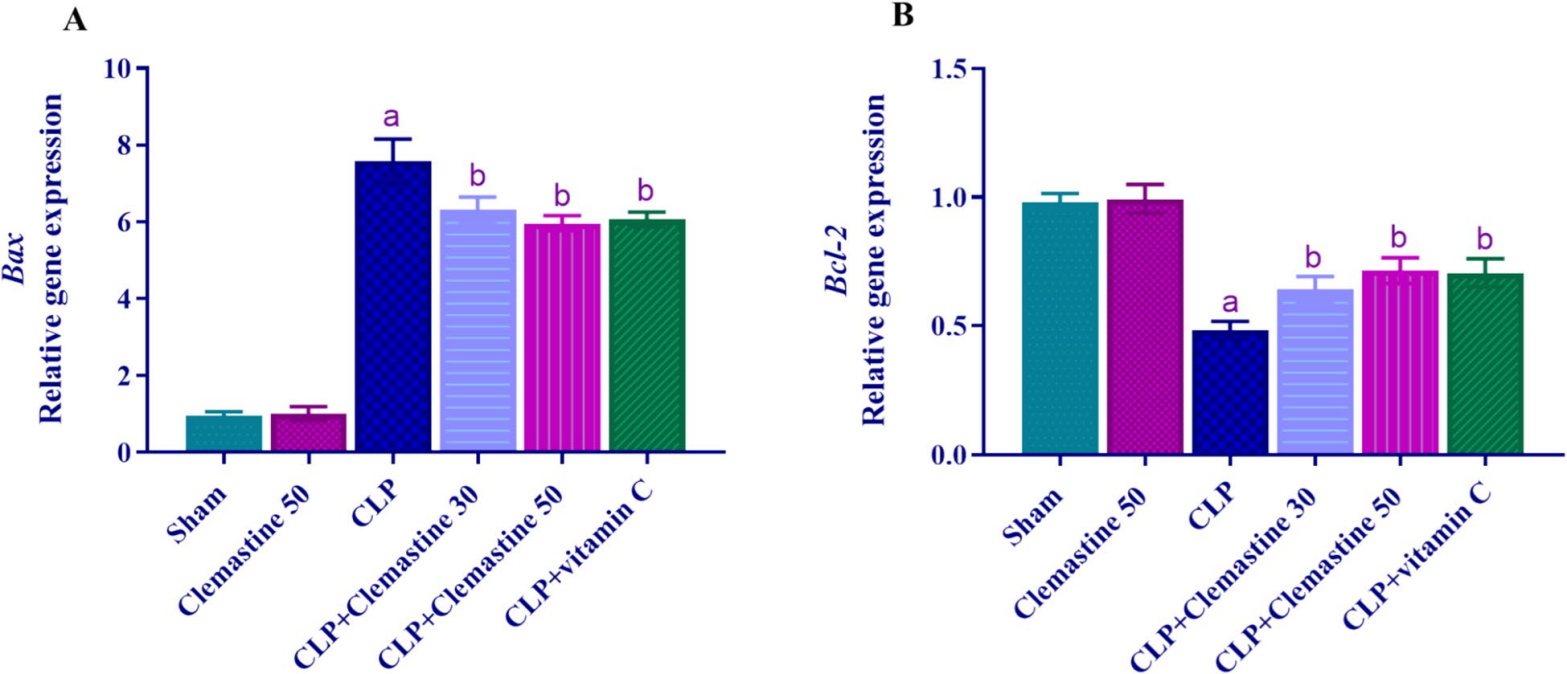
Clemastine effect on **A** *Bax* and **B** *Bcl-2* hepatic mRNA levels in septic liver damage. Expression was normalized to GAPDH gene expression and shown relative to the sham group. Each value is represented as the mean ± standard deviation. (n = 6). Level of significance: a; p < 0.05 in contrast to sham rats, b; p < 0.05 in contrast to CLP rats, and c; p < 0.05 in contrast to CLP + Clemastine 30. *Bax* (Bcl-2)-associated X, *Bcl-2* B-cell lymphoma-2

The hepatic mRNA expression levels of *Bax* and *Bcl-2* were evaluated to determine the effect of CLM on the regulation of apoptosis. As depicted in Fig. 8A, B, it is evident that CLP significantly increased the expression of the *Bax* gene while concurrently reducing the expression of the *Bcl-2* gene (p < 0.05) when compared to the sham rats. In contrast, the expression of the *Bax* gene was significantly diminished (p < 0.05) in both the CLM-treated rats and the CLP + vitamin C rats, whereas the expression of the *Bcl-2* gene was notably increased (p < 0.05) in comparison to the CLP group.

### Clemastine diminished *NF-kB* and *caspase-3* gene expression

**Fig. 9:**
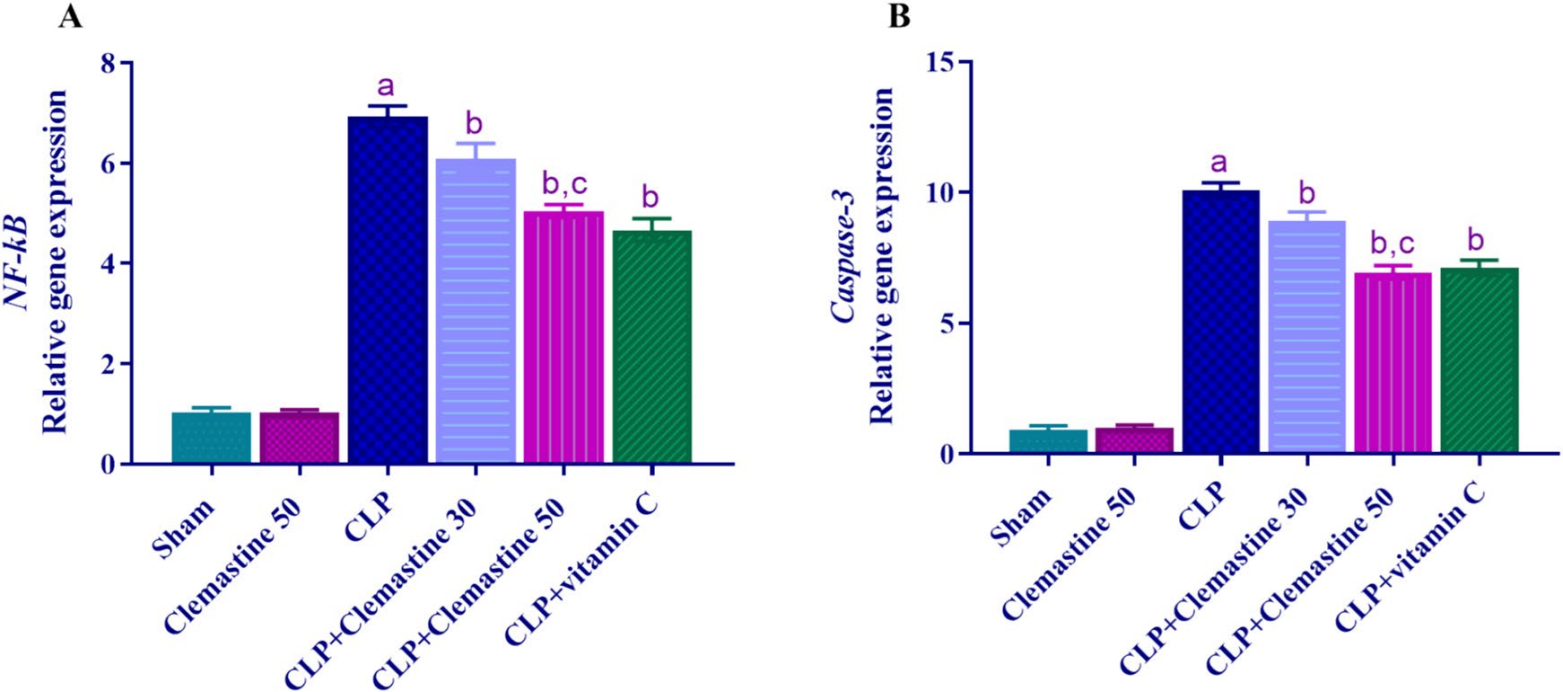
Clemastine effect on **A** *NF-kB* and **B** *caspase-3* hepatic mRNA levels in septic liver damage. Expression was normalized to GAPDH gene expression and shown relative to the sham group. Each value is represented as the mean ± standard deviation. (n = 6). Level of significance: a; p < 0.05 in contrast to sham rats, b; p < 0.05 in contrast to CLP rats, and c; p < 0.05 in contrast to CLP + Clemastine 30. *NF- kB* nuclear factor-kappa B

CLM's anti-apoptotic impact in sepsis was evaluated by measuring the hepatic mRNA expression of the *NF-kB* and *caspase-3* genes. The hepatic mRNA levels of the *NF-kB* and caspase-3 genes were dramatically upregulated (p < 0.05) in the CLP rats compared to the sham rats. In contrast to CLP rats, the administration of CLM caused a significant dose-dependent decrease (p < 0.05) in the expression of the *NF-kB* and *caspase-3* genes in the liver (Fig. 9A, B).

### Clemastine regulated NLRP-3, caspase-1, GSDMD c-NT, NF-KB p-P65 and cleaved caspase-3 protein expression

To assess the CLM's anti-pyroptotic impact, the hepatic levels of NLRP-3, caspase-1, GSDMD c-NT, NF-KB p-P65 and cleaved caspase-3 proteins were analyzed using Western blotting.

**Fig. 10:**
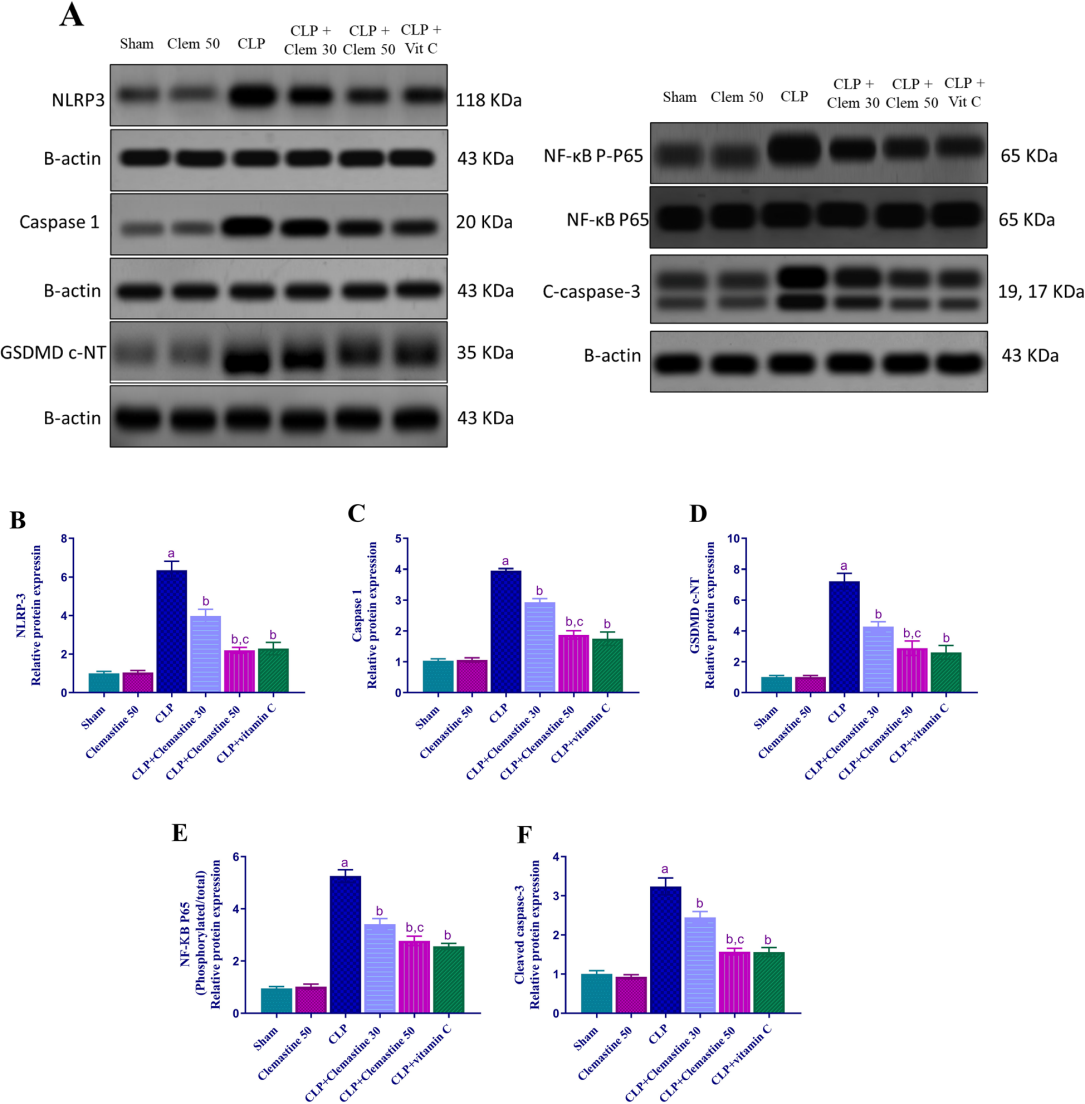
Clemastine effect on NLRP-3, caspase-1, GSDMD c-NT, NF-KB p-P65 and cleaved caspase-3 protein levels. **A** Representative western blots of NLRP-3, caspase-1, GSDMD c-NT, NF-KB p-P65, cleaved caspase-3 and β-actin proteins. **B**–**F** Following the normalization of the bands in panel **A** to the relevant internal control, β-actin, densitometric analysis was employed to quantify protein expressions as a fold change relative to the sham rats. Each value is represented as the mean ± standard deviation (n = 3). Level of significance: a; p < 0.05 in contrast to sham rats, b; p < 0.05 in contrast to CLP rats, and c; p < 0.05 in contrast to CLP + Clemastine 30. *NLRP-3* NOD-like receptor family pyrin domain containing 3, *GSDMD c-NT* cleaved gasdermin D N-terminal domain

As depicted in Fig. 10A–F, there is a dramatic up-regulation (p < 0.05) of the hepatic NLRP-3, caspase-1, GSDMD c-NT, NF-KB p-P65 and cleaved caspase-3 proteins of CLP rats, after the adjustment of band magnitudes to the internal control β-actin, in comparison to sham rats. Contrariwise, CLM administration revealed a significant dose-dependent expression suppression (p < 0.05) of these proteins relative to untreated rats.

### Clemastine prevented alteration in hepatic histopathology

**Fig. 11:**
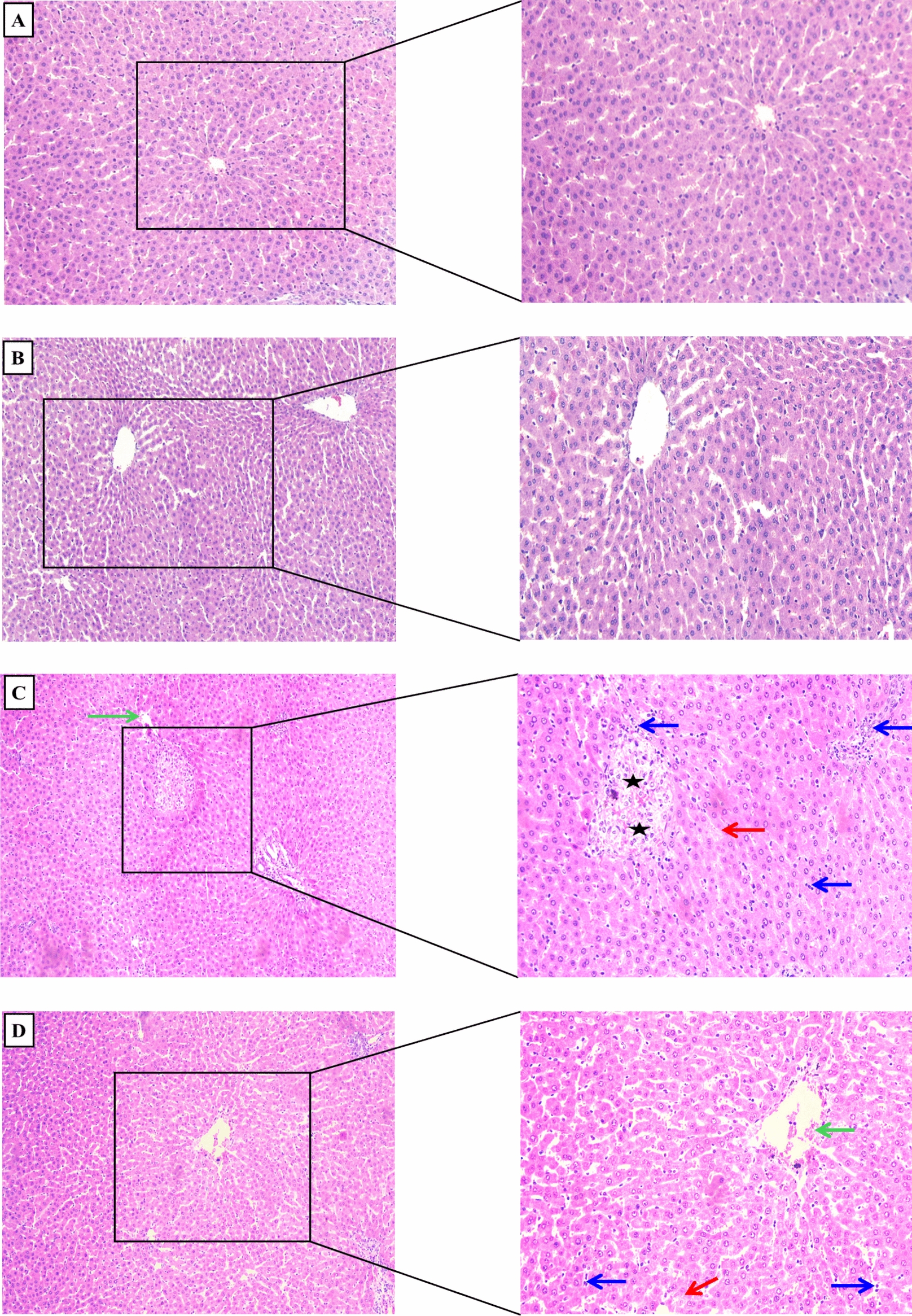

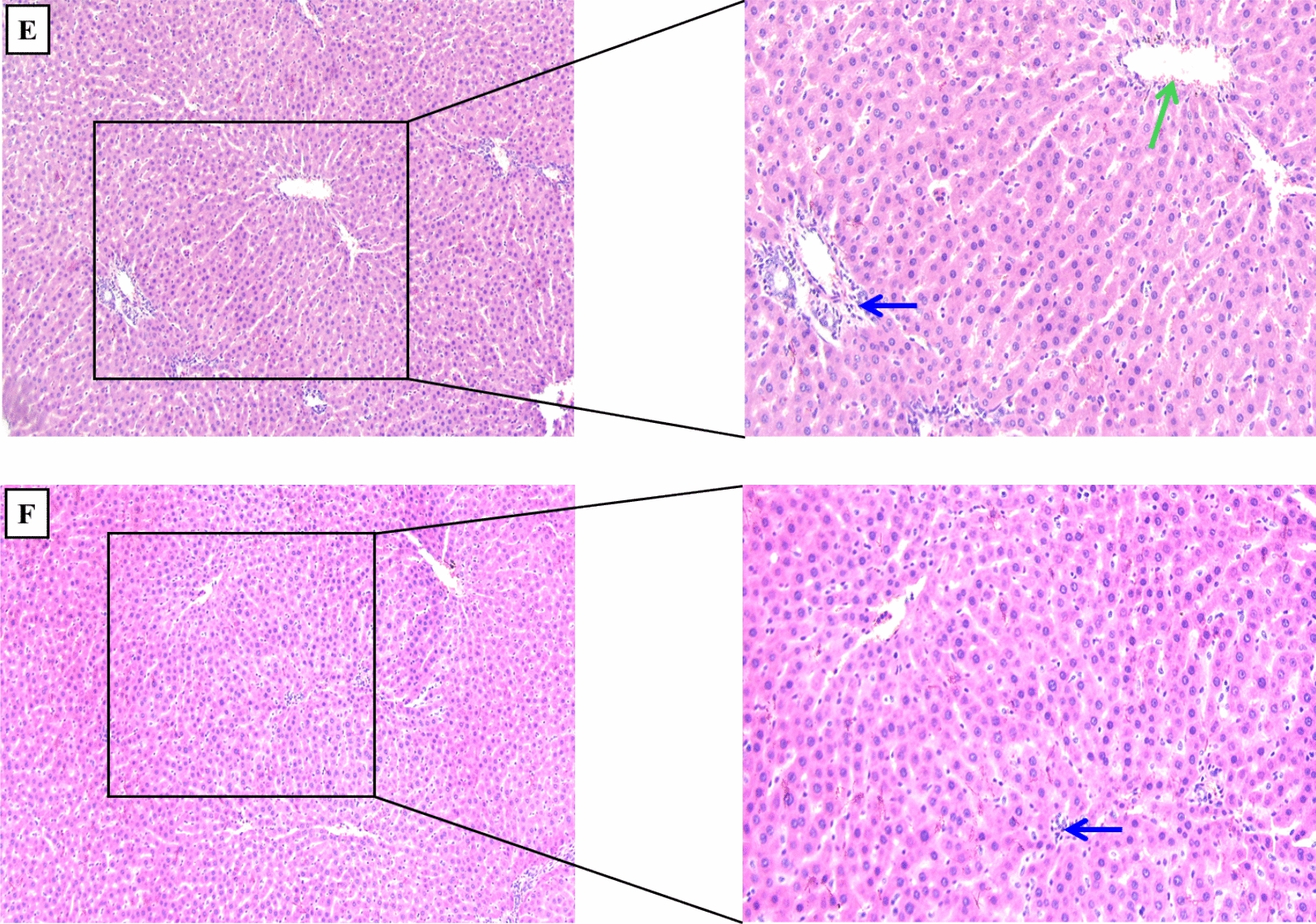
Histopathological alterations in hepatic tissues. Hepatic tissue images of different groups (H&E staining, × 100, × 200). **A** Sham, **B** Clemastine 50, **C** CLP, **D** CLP + Clemastine 30, **E** CLP + Clemastine 50, and **F** CLP + vitamin C. Blue arrow: inflammatory cell infiltration, green arrow: vascular congestion, red arrow: hepatocyte ballooning, black star: hepatocyte necrosis

As depicted in Fig. 11A–F and Table 2, The hepatic tissues of septic rats revealed a significant elevation (p < 0.05) in inflammatory cell infiltration, vascular congestion, hepatocyte ballooning, and necrosis in contrast to sham group that depicted normal hepatic tissues. However, treatment with CLM showed a marked (p < 0.05) dose-dependent mitigation of these abnormalities. Additionally, treatment with CLM 30 mg/kg exhibited minimal periportal inflammatory cell infiltrate, moderate hepatocyte ballooning, mild necrosis, and mild vascular congestion, as depicted in Fig. 11D. Moreover, CLM high dose illustrated minimal hepatocyte necrosis and inflammation with absent ballooning, as presented in Fig. 11E.

**Table 2:** Hepatic histological scoring

Groups	Sham	Clem 50	CLP	CLP + Clem 30	CLP + Clem 50	CLP + Vit C
Inflammatory cell infiltration	0	0	3	2	1	1
Vascular congestion	0	0	2	1	1	0
Hepatocyte ballooning	0	0	3	2	0	0
Hepatocyte necrosis	0	0	3	1	1	1
Total scoring	0	0	11^a^	6^b^	3^b, c^	2^b^

Level of significance: a; p < 0.05 in contrast to sham rats, b; p < 0.05 in contrast to CLP rats, c; p < 0.05 in contrast to CLP + Clemastine 30

## Discussion

Sepsis is a devastating clinical condition influencing critically ill individuals globally with an annual frequency exceeding 48 million cases and resulting in approximately 11 million sepsis-related fatalities, alarmingly, these figures are doubling each year [6]. Sepsis is a complicated group of conditions that induces tissue injury not only through the excessive production of ROS but also through the activation of exacerbated local and systemic inflammatory responses that result in multiple organ damage [54, 55].

The CLP model is regarded as the most reliable method for the induction of sepsis, as it can lead to significant peritonitis and multi-organ failure due to its close resemblance to the pathological manifestations of sepsis observed in clinical settings [56]. Utilizing a CLP rat model, this study attempted to examine whether pretreatment with CLM might produce protection against septic liver impairment for the first time, in addition to its documented cardioprotective positive outcomes in sepsis [34].

In light of the elevated mortality rate linked to sepsis, we began our study by examining the impact of CLM on the survival of septic rats. Our results indicated a 45% death rate from sepsis on the second day and a 30% mortality rate on the third day post-surgery. Fortunately, we established that early injection of CLM prior to sepsis induction led to a substantial decrease in mortality to 20% by the second postoperative day.

The liver is the major organ in preserving metabolic equilibrium and generating strong host defenses against infections [15, 57, 58]. Liver failure is an early indicator of a bad prognosis in people with sepsis [59]. Liver damage is induced because of pathogens and toxins during sepsis. The injury exaggerates from active hepatocellular malfunction to hepatic impairment and eventually to hepatic failure [15]. Sepsis continues to pose a significant challenge for medical researchers and practitioners owing to its intricate pathophysiology.

The potential measure of the systemic inflammatory reaction to infection is PCT, which has become an early sepsis diagnosis marker recently [60]. PCT expression dramatically elevated during sepsis and peaked between 6 and 24 h later [61]. The present study showed elevated levels of PCT after CLP which have attenuated after Clemastine treatment, indicating its possible protection against sepsis.

Moreover, the CLP technique was proven to result in sepsis, with additional liver dysfunction and hepatic damage within 1.5 h [62]. The most widely used hepatic markers, ALT and AST showed that CLP was hepatotoxic, whereas higher levels represented the early reaction to liver damage [63, 64]. Al-Kadi et al. have recorded a deterioration of liver function in the first phases of sepsis, terminating in hepatic failure in rats [65]. It has been proven that increased ALT and AST activities are associated with hepatic inflammation and injury [66, 67]. In this investigation, CLM's hepatic protective influence was shown by a marked dose-dependent decrease of ALT and AST serum levels which was further confirmed by histopathological improvement in the rats given CLM. The histological evaluation of liver tissue integrity indicated a significant improvement in damage and a restoration of the overall lobular architecture of the hepatic tissue. This finding suggests that CLM has positively influenced liver function and mitigated hepatic injury.

Furthermore, CLM has been documented to dramatically reduce oxidative stress in several tissues, including the heart and brain [34, 35, 68]. The current investigation showed that the antioxidant markers GSH and SOD were reduced after sepsis; meanwhile, they were dramatically raised following the administration of CLM, indicating the antioxidant benefit of CLM. Moreover, it has been shown that MDA, which is generated as a result of fatty acid peroxidation caused by reactive oxygen species, promotes apoptosis [69, 70]. The current findings depicted that the untreated rats had significantly elevated levels of hepatic MDA. Nevertheless, CLM treatment resulted in dramatic dose-dependent suppression of MDA levels, indicating that CLM may have a strong impact in attenuating oxidative stress during sepsis.

Moreover, sepsis induction leads to the generation of proinflammatory cytokines such as IL-1β, IL-18, and TNF-α [71]. This is in line with our findings, which demonstrated that CLM had a dose-dependent inhibitory impact on the high levels of these cytokines during sepsis, hence suggesting the anti-inflammatory effect of CLM in sepsis.

Hoping for a more comprehensive elucidation of the protective mechanism by which CLM mitigates liver dysfunction induced by CLP. Protein levels of NLRP-3, Caspase-1, and GSDMD C-NT were analyzed. NLRP-3 s are intracellular proteins implicated in the immune system in mammals. Their overexpression largely influences sepsis [25, 72, 73]. Additionally, previous studies have linked hepatic pyroptosis to liver damage caused by the CLP process [64, 74].

Numerous investigations have demonstrated the role of NF-κB in NLRP-3 activation during sepsis that aggravates the inflammatory reaction toward septic shock [75, 76].

NLRP-3 combines with apoptosis-associated speck-like protein and activates procaspase-1 to produce an inflammatory complex. Following the inflammatory complex's cleavage of procaspase-1, mature IL-1β and IL-18 are produced by caspase-1. Additionally, caspase-1 proteolyzes GSDMD into cleaved GSDMD, which forms large plasma membrane pores and further triggers pyroptosis [77, 78]. In addition, Fu, Yanbin, et al. have documented that the activation of NLRP-3 was blocked by the Klotho protein, recognized as a biomarker for anti-aging, is a humoral factor identified within the circulatory system [79, 80]. In a similar manner, our findings indicated that the downregulation of klotho following sepsis was ameliorated by CLM, resulting in the inactivation of NLRP-3.

Furthermore, Aboyoussef AM et al. have reported increased protein levels of NLRP-3, IL-1β, and caspase-1 after CLP [64]. Our findings were confirmed by these previous investigations which demonstrated that the CLP group had elevated levels of NF-κB, NLRP-3, Caspase-1, and GSDMD C-NT. Remarkably, NF-κB, NLRP-3, Caspase-1, and GSDMD C-NT revealed considerable dose-dependent expression downregulation in response to CLM indicating its anti-pyroptotic effect during sepsis-induced liver dysfunction. This is consistent with a report by Motawi, Tarek K., et al. that suggested the NLRP-3 signaling cascade as a possible explanation for CLM efficacy in attenuating autoimmune encephalomyelitis [36].

Apoptosis is dramatically promoted during sepsis because of the elevated expression of the *Bax/Bcl-2* ratio [81–83]. Additionally, *caspase-3* should be assessed as it is the primary apoptotic effector and its activation results in cell death [84, 85]. This is in line with our results that revealed the decreased *Bcl-2* gene expression, whereas the upregulated *Bax* and *caspase-3* genes expression after sepsis induction. Notably, the current study depicted that CLM markedly downregulated *Bax*, and *caspase-3* gene expression, and conversely stimulated the *Bcl-2* gene expression indicating its anti-apoptotic impact during sepsis. Previous investigations documented CLM's anti-apoptotic effect through stimulating Bcl-2 and suppressing Bax in cardiac damage caused by sepsis and myocardial ischemia–reperfusion Injury [34, 68].

Recent studies suggest that there is significant crosstalk between apoptotic and pyroptotic pathways, especially under inflammatory conditions such as sepsis. For example, caspase-3 (apoptotic) and caspase-1 (pyroptotic) can be activated simultaneously in response to severe cellular stress or infection [19, 86]. Moreover, under certain circumstances, caspase-3 can cleave GSDME, leading to a form of secondary pyroptosis, while caspase-1 activation can also influence apoptotic signaling [87, 88].

Our data demonstrated that CLM treatment mitigates both apoptotic (downregulation of Bax and caspase-3, upregulation of Bcl-2) and pyroptotic (inhibition of NLRP-3, caspase-1, and GSDMD) markers. This suggests that CLM exerts a broad cytoprotective effect, potentially by modulating upstream inflammatory signals such as NF-κB that drive both apoptotic and pyroptotic cell death. The dual inhibition may be particularly beneficial in the context of sepsis, where excessive cell death and inflammation contribute to organ dysfunction.

The present study elucidated the hepatoprotective effects of CLM in the setting of sepsis. Nevertheless, since sepsis results in various organs failure, more studies in the future are necessary to evaluate other organs'functions. While we assessed molecular and biochemical markers of pyroptosis and apoptosis in liver tissue, incorporating in vitro assays or imaging-based validation (such as immunofluorescence or TUNEL staining) would strengthen the mechanistic conclusions and provide more direct evidence of the pathways involved. We propose that future studies should include in vitro assays (e.g., hepatocyte cultures with inflammasome activation) and use of pathway-specific inhibitors.

## Conclusion

In conclusion, CLM resulted in a dramatic dose-dependent alleviation of liver injury caused by CLP, via attenuating oxidative stress, apoptosis, and NLRP-3/Caspase-1 mediated pyroptosis. The current findings depicted that CLM markedly inhibited oxidative damage and inflammation in the liver because of sepsis via upregulating levels of KLA, GSH, and SOD and suppressed those of PCT, IL-18, MDA IL-1β, and TNFα, Furthermore, it mitigated apoptosis via downregulation of *Bax*, *NF-kB*, and *caspase 3* genes while elevating *Bcl-2* gene expression. Additionally, CLM repressed pyroptosis by downregulating the expression of NLRP-3, caspase-1, and GSDMD c-NT proteins. Consequently, this investigation indicates the possibility that CLM when used as a pre-anesthetic medication, might lower postoperative sepsis incidence; more studies are necessary to validate this positive impact in humans. Moreover, future studies should evaluate the timing, dosing, and safety profile of CLM in clinically relevant models.

## Data Availability

All data produced or examined during this investigation are incorporated in this published article.
